# Do Sex and Gender-Related Differences Account to Different Risk of Developing Heart Failure in Middle-Aged People with Metabolic Syndrome?

**DOI:** 10.3390/metabo14100528

**Published:** 2024-09-30

**Authors:** Stefano Bonapace, Alessandro Mantovani

**Affiliations:** 1 Division of Cardiology, IRCCS Ospedale Sacro Cuore Don Calabria, 37024 Negrar di Valpolicella, Italy; 2 Department of Medicine, Section of Endocrinology, Diabetes and Metabolism, University of Verona, 37100 Verona, Italy; alessandro.mantovani@univr.it

Metabolic syndrome (MetS) is not a disease but a constellation of metabolic abnormalities that together increase the risk of developing cardiovascular disease (CVD) [1]. The National Cholesterol Education Program Adult Treatment Panel III (NCEP ATP III) defines metabolic syndrome as the presence of three or more of the following conditions: abdominal obesity (waist circumference >102 cm for men and >88 cm for women), elevated fasting glucose (≥100 mg/dL) or a diagnosis of diabetes, high blood pressure (≥130/85 mm Hg or use of antihypertensive medication), elevated triglycerides (≥150 mg/dL), and reduced high-density lipoprotein (HDL) cholesterol (<40 mg/dL in men, <50 mg/dL in women) [2]. In recent decades, the prevalence of MetS has dramatically increased, particularly among young adults and adolescents, representing a significant public health concern [3]. One of the most alarming consequences of metabolic syndrome is its strong association with cardiovascular diseases, particularly heart failure (HF). HF is considered a pandemic, affecting an estimated 64 million people worldwide [4], and its prevalence will increase due to the aging of the population. Most recent projections for the US suggest an increase in the prevalence of HF by about 46% from 2012 to 2030, with a corresponding increase in healthcare costs of about 127% [5]. The consensus document of the various international scientific societies dealing with HF proposed a universal definition of HF as a “clinical syndrome with symptoms and/or signs caused by a structural and/or functional cardiac abnormality corroborated by elevated natriuretic peptide levels and/or objective evidence of pulmonary or systemic congestion” [6]. They also proposed a new and revised classification of symptomatic HF according to left ventricular ejection fraction (LVEF). This includes HF with reduced ejection fraction (HFrEF, LVEF ≤ 40%); HF with mildly reduced ejection fraction (HFmrEF, LVEF 41–49%); and HF with preserved ejection fraction (HFpEF, LVEF ≥ 50%). Additionally, a new entity, HF with improved LVEF (HFimpEF with baseline LVEF ≤ 40%, a ≥10 point increase from baseline LVEF, and a second measurement of LVEF > 40%), was introduced to account for the dynamic and changing clinical scenario of HF syndrome where LVEF improves with treatment [6]. While heart failure has traditionally been considered a condition of older adults, emerging evidence highlights its increasing occurrence in younger populations, particularly among individuals with metabolic syndrome [7,8]. In fact, epidemiological data from Europe and North America indicate a decline in the age-specific incidence of HF, with a progressive shift towards HF with preserved ejection fraction among women, suggesting an evolving epidemiology of HF [9]. Moreover, age-adjusted mortality in young adults (aged 15–44 years) increased from 2.36 in 1999 to 3.16 in 2019, a greater rise than in older adults (aged ≥ 75 years) [9]. Therefore, this intersection between metabolic syndrome and heart failure in young adults is a growing concern, given the lifelong burden and disability that it imposes on individuals and healthcare systems [5]. Physiopathologically, the different components of metabolic syndrome may affect left ventricular function through various hemodynamic and neurohormonal non-hemodynamic mechanisms [10,11]. Hypertension and obesity produce chronic hemodynamic pressure and volume overload that impact myocardial hypertrophy and fibrosis. Hypertension, obesity, hyperglycemia, and increased visceral fat determine neurohormonal changes inducing insulin resistance, activating the renin-angiotensin system (RAAS) as well as the sympathetic nervous system, favoring oxidative stress, and changing myocardial free fatty acid (FFA) energetic metabolism at the mitochondrial level, leading to lipotoxicity [12]. In addition, endothelial and microvascular dysfunction are consequences of MetS. All these hemodynamic and neurohomonal/metabolic changes may promote myocardial apoptosis and interstitial fibrosis [10,11,12] that together with hypertrophy, increase left ventricular (LV) myocardial stiffness and LV filling pressure, leading to diastolic dysfunction and HFpEF [10,11].

In their paper, recently published in *Metabolites*, Kim et al. [13] retrospectively investigated metabolic status as a risk factor for HF in a large population of males and females in their 40s using nationwide real-world data obtained from the Korean National Health Insurance Service from 2009 to 2016, thus expanding their previous observations in middle-aged subjects between 50 and 59 years of age [14]. Subjects were divided into three groups (normal, pre-MetS with one or two components of MetS, and MetS). They found that MetS increased the risk of HF by 1.97-fold in males and by 2.40-fold in females and that pre-MetS increased the risk by 1.61-fold in males and by 1.89-fold in females, further confirming the higher risk in females than in males observed earlier [14], but with an even higher hazard ratio at these younger ages [13]. These data corroborate recent observations that the increasing trend in HF among younger populations is associated with a rise in metabolic burden in this group compared to older subjects [15].

However, the main scenario highlighted by the study is the higher risk of developing HF in females compared to males. Earlier studies showed that not only sex-specific differences but also gender-specific (i.e., psycho–socio–cultural factors) differences play a significant role in the presentation and progression of metabolic syndrome [16,17], and this might also influence the different risk of developing HF in males and females. The authors confirmed a different prevalence of MetS in the two groups, higher in males (18.35%) and lower in females (5.21%), but females with MetS showed a higher risk for developing HF [10]. McNeill et al. reported that men and women with MetS were approximately 1.5 and 2 times more likely to develop CVD than control subjects after adjustment for age, smoking, LDL cholesterol, and race [18].

Why should Mets and pre-MetS expose females to a higher risk of developing HF compared to males? Do the single elements of MetS have the same weight in developing HF in males and females? The components of MetS, including obesity, insulin resistance, hypertension, and dyslipidemia, manifest differently in men and women [19], and they could probably weigh differently in the risk of developing heart failure between sexes. In other words, in a male and a female with three components of MeTs, i.e., hypertension, hyperglycemia, and high triglycerides, the relative weights of single elements on the incidence of HF might not be the same because of sex-specific differences.

Although the study cannot clarify the physiopathological reasons for these differences, it opens various hypotheses. What was the hormonal status of the female group? How many females were premenopausal and postmenopausal in the considered ages? Are behavioral gender-specific differences accounting for the different impact of developing HF?

Men are more likely to develop central or visceral obesity, which is characterized by the accumulation of fat around the abdomen and liver [20]. This type of fat is more metabolically active and is associated with a higher risk of insulin resistance, dyslipidemia, and cardiovascular disease, particularly HF [21]. Of note in this study, the authors observed that in females, a slight elevation of alanine-aminotransferase (ALT) levels (40–99 IU/L) increased the risk of HF by 2.1-fold, but not in males. Premenopausal women tend to accumulate fat subcutaneously, particularly around the hips and thighs. This pattern of fat distribution, known as “pear-shaped” obesity, is considered less harmful than visceral fat [20]. However, after menopause, women tend to experience a shift toward central obesity, increasing their risk of MetS and CVD [22]. Women generally maintain better insulin sensitivity than men during their reproductive years, which may offer some protection against the early development of cardiovascular diseases [22]. However, postmenopausal women experience a decline in insulin sensitivity, increasing their risk of MetS and CVD [22]. Premenopausal women generally have lower blood pressure than men, likely due to the protective effects of estrogen [23]. However, after menopause, blood pressure increases significantly in women, narrowing the gender gap in hypertension prevalence [23]. Premenopausal women are more likely to have higher HDL cholesterol levels than men, but this protective factor diminishes after menopause [24]. Postmenopausal women with MetS often exhibit a more atherogenic lipid profile, similar to that of men [24]. The loss of estrogen’s anti-inflammatory effects may exacerbate inflammation and oxidative stress [22]. This hormonal shift may accelerate the development of heart failure in MetS and pre-MetS women compared to males, justifying the sex-related difference observed by the authors. We can also speculate that another reason for this higher risk of developing HF in females with MetS compared to males may be related to a different phenotype of HF [9], although the authors do not have any information about this. Males usually develop HF with reduced ejection fraction (HFrEF), whereas women have a higher incidence of HF with preserved ejection fraction related to an impaired left ventricular filling with a normal pump function [9]. Reduced estrogens may favor cardiac fibrosis and alter cardiac energy metabolism, which may render the left ventricle stiffer and more prone to developing diastolic dysfunction [25].

It would be interesting and a reason for future research if the authors were able to trace through the data from the Korean National Health Insurance Service the state of premenopause and postmenopause in these patients and run an analysis with this variable to evaluate its impact on the risk of developing heart failure in the two groups.

An additional interesting observation found in this manuscript is the different impact of smoking as a risk factor for developing heart failure in the two sexes. Current smoking increased the risk 2.4-fold in females but not in males [10]. The fact that there is a different impact on the risk of developing HF related to smoking suggests that there are not only sex-specific differences related to hormones but also gender-specific differences related to different social behaviors.

The intersection of metabolic syndrome and heart failure in middle-aged subjects represents a growing public health challenge. The rising prevalence of metabolic syndrome in this age group, coupled with the early onset of heart failure, has significant implications for long-term health outcomes, and the sex/gender differences observed render it more complex to deal with. The pathophysiological mechanisms linking MetS and HF—insulin resistance, obesity, hypertension, dyslipidemia, and chronic inflammation—are well-established but present differently in men and women, underscoring the need for increased awareness in health professionals to identify these differences for early management and intervention. Despite a growing body of evidence, the relative contributions of sex in terms of biological, genetic, and hormonal differences and gender in terms of sociocultural dimensions to the manifestations and outcomes of heart failure in MetS remain unknown and need to be elucidated. Gender and sex-related factors are important contributors to health disparities and disease outcomes, and certain sex/gender-related factors may be difficult to modify. By addressing modifiable risk factors through lifestyle changes, appropriate medical management, and also cultural changes, the progression of both metabolic syndrome and heart failure in these individuals can be mitigated, ultimately reducing the burden of cardiovascular disease across the lifespan.
